# The precision of robotic surgery in International Brazilian Journal of Urology

**DOI:** 10.1590/S1677-5538.IBJU.2022.02.01

**Published:** 2022-03-01

**Affiliations:** 1 Universidade do Estado do Rio de Janeiro Unidade de Pesquisa Urogenital Rio de Janeiro RJ Brasil Unidade de Pesquisa Urogenital - Universidade do Estado do Rio de Janeiro - Uerj, Rio de Janeiro, RJ, Brasil; 2 Hospital Federal da Lagoa Rio de Janeiro RJ Brasil Serviço de Urologia, Hospital Federal da Lagoa, Rio de Janeiro, RJ, Brasil

The March-April number of Int Braz J Urol, the 15th under my supervision, presents original contributions with a lot of interesting papers in different fields: Robotic Surgery, Prostate Cancer, Male Infertility, BPH, Overactive Bladder, Undescended Testis, Adrenal Adenomas, Enuresis, UPJ obstruction, Urethral Strictures, Penile cancer and Hypospadia. The papers came from many different countries such as Brazil, USA, China, Italy, Colombia, Lithuania, Turkey, Spain, France, UK and Poland, and as usual the editor´s comment highlights some of them.

In the present issue we present important papers about robotic prostatectomy of the group of Dr. Patel from USA (1–3). The paper and the 2 videos shows the robotic surgery advantages in terms of operative view magnification, potency outcomes following robotic-assisted radical prostatectomy and described the current Robotic-assisted Radical Prostatectomy technique with details and considerations regarding the optimal approach to neurovascular bundle preservation after performing 15,000 cases, a very expressive experience. This important paper is the cover in this edition. The editor in chief would like to highlight the following works too:

Dr. Bakavicius and colleagues from Lithuania, USA, UK and Brazil, presented in page 263 (4) a nice systematic review about the HIFU for the focal treatment of prostate Cancer and concluded that six months after focal HIFU therapy 98% of patients were totally continent and 80% of patients retained sufficient erections for sexual intercourse. The majority of complications presented in the early postoperative period and were classified as low-grade.

Dr. Truzzi and colleagues from Brazil presented in page 220 (5) a important update about the concepts and treatment of neurogenic bladder and concluded that the treatment guidelines are important tools for the public health system to promote the best practice when treating neurogenic bladder patients.

Dr. Sum and colleagues from China presented in page 275 (6) a nice study about the association between winter season and desmopressin treatment efficiency in children with monosymptomatic nocturne enuresis (MNE) and concluded that winter season and severity of symptoms are two risk factors associated with desmopressin treatment failure in MNE patients.

Dr. Jia and colleagues from China presented in page 303 (7) a cross-sectional study about the risk factors of inguinal lymph node metastasis in patients with penile cancer and concluded that the maximum inguinal lymph node (ILN) diameter, pathological T stage, pathological differentiation, and lymphatic vascular infiltration were independent risk factors for ILN metastases.

Dr. Özçift and colleagues from Turkey presented in page 316 (8) a nice sudy about the relationship between vitamin D status and Overactive bladder (OAB)-related symptoms and quality of life (QoL) in children and concluded that Vitamin D deficiency is more common in children with urinary incontinence and OAB than in healthy children. Although vitamin D deficiency is not routinely evaluated for every patient, it should be evaluated in treatment-resistant OAB cases. Vitamin D supplementation may improve urinary symptoms and QoL in patients with OAB.

Dr. Bertolo and colleagues from Italy presented in page 328 (9) a nice study about the Thulium laser enucleation of prostate (ThuLEP) versus laparoscopic trans-vesical simple prostatectomy (LSP) in the treatment of benign prostatic hyperplasia (BPH) and concluded that LSP and ThuLEP are comparable in relieving from BPO and improving the patient-reported outcomes. Invasiveness of LSP is more significant.

The Editor-in-chief expects everyone to enjoy reading.
